# Enzalutamide versus flutamide for castration-resistant prostate cancer after combined androgen blockade therapy with bicalutamide: study protocol for a multicenter randomized phase II trial (the OCUU-CRPC study)

**DOI:** 10.1186/s12885-019-5526-3

**Published:** 2019-04-11

**Authors:** Taro Iguchi, Satoshi Tamada, Minoru Kato, Sayaka Yasuda, Takeshi Yamasaki, Tatsuya Nakatani

**Affiliations:** Department of Urology, Osaka City University Graduate School of Medicine, 1-4-3 Asahi-machi, Abeno-ku, Osaka, 545-8585 Japan

**Keywords:** Castration-resistant prostate cancer, Enzalutamide, Flutamide

## Abstract

**Background:**

Enzalutamide is an oral androgen receptor targeted agent that has been shown to improve survival in PREVAIL trials and has been approved for patients with chemo-naïve metastatic castration-resistant prostate cancer (CRPC). Meanwhile, flutamide is a non-steroidal oral anti-androgen that was commonly used before the approval of bicalutamide. The objective of the OCUU-CRPC study is to compare the efficacy and safety between second-line hormonal therapy of enzalutamide and flutamide as alternative anti-androgen therapy (AAT) after combined androgen blockade (CAB) therapy that included bicalutamide in patients with CRPC.

**Methods:**

A total of 100 patients with CRPC with or without distant metastases after disease progression who received CAB therapy with bicalutamide were randomly assigned at a 1:1 ratio according to distant metastases to the enzalutamide (160 mg/day, 4 × 40 mg capsules once daily) and flutamide (375 mg/day; 3 × 125 mg tablets thrice daily) groups. The primary endpoint for the drug efficacy is the response rate of prostate-specific antigen (PSA) (i.e., the ratio of patients whose PSA declined by ≥50% from baseline) at 3 months. Meanwhile, the secondary endpoints are PSA progression rate at 3 and 6 months, PSA response rate at 6 months, change in quality of life, PSA progression-free survival, and safety. The patient registration started in January 2015 and will end in March 2018, and the follow-up period is 6 months after the last patient registration. The main result will be reported in March 2019.

**Discussion:**

In the OCUU-CRPC study, we compare the efficacy and safety of enzalutamide or alternative AAT with flutamide in participants with CRPC who were previously treated with a CAB therapy with bicalutamide. The expected results of this study will be that enzalutamide is superior to flutamide in terms of PSA response. A longer time to disease progression with enzalutamide over flutamide may translate to better overall survival. However, flutamide may be more accessible for patients owing to its lower cost than enzalutamide.

**Trial registration:**

The OCUU-CRPC study was prospectively registered at clinicaltrials.gov (NCT02346578, January 2015) and University Hospital Medical Information Network (UMIN000016301, January 2015).

## Background

Prostate cancer is the most commonly diagnosed cancer and the third leading cause of cancer-related death among men worldwide [1]. In most patients who are treated for advanced recurrent prostate cancer with androgen-deprivation therapy (ADT) that comprise a luteinizing hormone-releasing hormone (LHRH) analogue or orchiectomy with or without an anti-androgen, disease progression occurs despite effective suppression of serum testosterone. These patients are then diagnosed with castration-resistant prostate cancer (CRPC).

In Japan, ADT has been widely used not only for advanced recurrent prostate cancer, but also for localized prostate cancer in elderly patients. Although ADT is effective only for a certain period and causes recurrence as CRPC, several patients actually respond to treatment for long periods [2]. Combined androgen blockade (CAB) therapy using an LHRH analogue with an anti-androgen is superior to ADT without an anti-androgen in terms of long-term efficacy among Japanese patients with prostate cancer [3]. Despite some disadvantages of CAB such as higher cost compared to ADT without anti-androgen, an observational study that compared CAB and castration showed good prognosis in patients with T1c-T3 prostate cancer [4]. At present, CAB using bicalutamide is more widely used in Japan than LHRH analogue monotherapy, and many elderly patients with localized prostate cancer are treated with CAB.

However, a proportion of patients with prostate cancer who are treated with CAB experience prostate-specific antigen (PSA) recurrence and are diagnosed as CRPC after confirming for anti-androgen withdrawal syndrome (AWS). Alternative anti-androgen therapy (AAT) with flutamide as the subsequent therapy after CAB therapy with bicalutamide is widely used before the androgen receptor targeted therapy (ART) era, particularly in Japan [5–11]. The response rate of AAT, defined as a decrease of > 50% from the baseline serum PSA level, was 22%, and patients who respond to AAT have been reported to have good prognosis [6]. This phenomenon is attributed to the different mechanism of actions among anti-androgens [9].

Flutamide, a non-steroidal oral anti-androgen, was often used in clinical practice before bicalutamide was approved. Some small, single-arm non-randomized studies suggest a PSA benefit in flutamide as second-line hormonal therapy [5–11]. However, the use of flutamide is optional for limited patients with CRPC according to the American Urological Association guidelines [12] owing to the modest PSA benefit, with PSA declines of > 50% occurring typically in only 20–40% of men with a median duration measured in several months.

Enzalutamide is an androgen receptor inhibitor that targets several steps in the androgen receptor signaling pathway. It inhibits binding of androgens to the androgen receptor, androgen-receptor nuclear translocation, and androgen receptor-mediated DNA binding [13]. In preclinical studies, enzalutamide showed a higher affinity for the androgen receptor and superior suppression of key components of the androgen receptor signaling pathway than bicalutamide [13, 14]. Subsequently, enzalutamide was approved for the treatment of metastatic CRPC based on the results of two pivotal placebo-controlled phase III trials, namely, AFFIRM [15] and PREVAIL [16].

Before the ART era, the treatment options for CRPC are limited, and AAT with flutamide has been widely used in Japan. However, no clinical studies compared AAT with flutamide and enzalutamide as treatment modalities for CRPC. Creating clinical evidence of the superiority of enzalutamide to AAT (bicalutamide to flutamide) in the post-AWS setting in terms of safety and efficacy would be meaningful. To the best of our knowledge, the OCUU-CRPC study is the first randomized head-to-head trial of enzalutamide versus flutamide in patients with CRPC after CAB therapy with bicalutamide.

## Methods/design

### Aim, design, and setting of the study

This study aims to compare the efficacy and safety of enzalutamide or AAT with flutamide in patients with CRPC who were previously treated with CAB therapy with bicalutamide. The efficacy and safety of enzalutamide and AAT with flutamide will be evaluated, and the effective therapy against CRPC after treatment with CAB therapy with bicalutamide will be investigated.

**Fig. 1:**
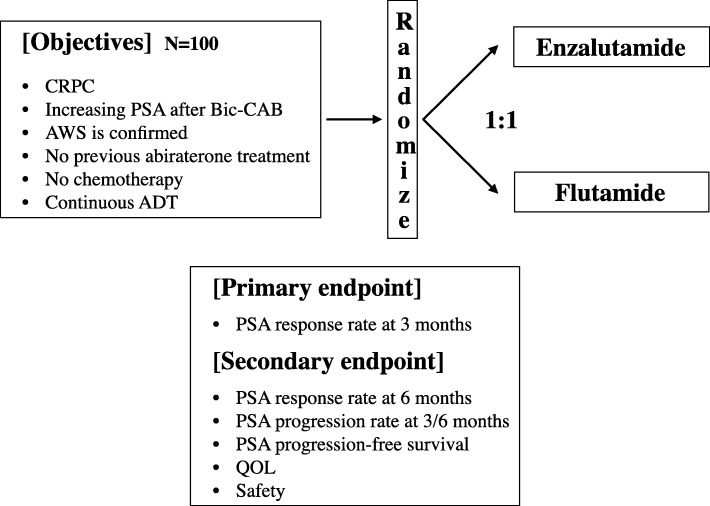
The design of the OCUU-CRPC study

The present study is a phase II, investigator-initiated, multicenter, open-labeled randomized clinical trial of enzalutamide and AAT with flutamide in patients with CRPC after treatment with CAB therapy with bicalutamide. Patients will be randomized to receive treatment with either enzalutamide or flutamide, as shown in Fig. 1.

### Participant characteristics

#### Study population

The study population consists of 100 patients with CRPC who were previously treated with CAB with bicalutamide and whose serum testosterone level is less than 50 ng/dL (1.73 nmol/L) and have progressive disease after confirmation of AWS. Disease progression is defined as at least one of the following criteria: PSA progression, soft-tissue disease progression, or bone disease progression according to the Prostate Cancer Working Group 2 criteria [17].

#### Eligibility criteria

The inclusion criteria are as follows:

1. Serum testosterone of less than 50 ng/dL
2. Disease progression diagnosed on imaging or PSA progression (i.e., consecutive increase of all PSA values measured at least thrice at a 1-week interval and a final value of 2 ng/mL or more. If the third value is not higher than the second one, a fourth measurement will be taken and its value must be higher than the second one in order for the patient to qualify)
3. Disease progression after CAB with bicalutamide
4. Eastern Cooperative Oncology Group performance status (PS) of 0 or 1
5. Age 20 years or older
6. Written informed consent

The exclusion criteria are as follows:

1. Any prior treatment with enzalutamide, flutamide, abiraterone, or chemotherapy, except for neoadjuvant therapy
2. Presence of active double cancer
3. Any prior treatment with bicalutamide within 6 weeks
4. Systemic biological therapy (except for existing approved drug as bone-modifying agents or treatment with LHRH analogues) or treatment with other antitumor agents for prostate cancer
5. Presence of severe complications
6. History of hypersensitivity to enzalutamide or any other excipient of enzalutamide
7. History of hypersensitivity to flutamide-containing agent
8. Liver dysfunction
9. Participants who are considered as ineligible by the investigator

#### Methods of recruitment and random allocation

Patient recruitment started in January 2015 and is targeted to end by March 2018. Eligible patients are randomly assigned to one of the two treatment groups through the data center at DOT International Inc. (which was responsible for data entry, coding, security, and storage, including any related processes to promote data quality). Patients will be randomly allocated to the enzalutamide or flutamide group via dynamic allocation using metastatic condition (M0, M1) and baseline PSA level as prognostic factors.

#### Treatment

Patients were randomly assigned at a 1:1 ratio to the enzalutamide (160 mg/day, 4 × 40 mg capsules once daily) or the flutamide (375 mg/day; 3 × 125 mg tablets thrice daily) group according to disease stage (M0 or M1). M0 means the absence of bone metastases on bone scan and of soft-tissue disease, while M1 means bone metastases on bone scan or soft-tissue metastases including nodal involvement above the aortic bifurcation. Both treatments are performed within the coverage of the National Health Insurance privilege of the patients. If the patients satisfy the criteria defined below for changing the drug, the initial medication used will be discontinued. The subsequent medication is not specified, and the investigator will choose the appropriate treatment option based on the patient condition. Enzalutamide is expected to be the primary drug for the subsequent treatment of patients in the flutamide group.

#### Criteria for changing the drug

The treatment will be changed in case of the following conditions:

1. Disease progression, as defined below (either of one)
  - PSA progression: Three consecutive increases in PSA, an increase of ≥25%, and an absolute increase of ≥2 ng/mL above baseline
  - Disease progression as noted on radiography
  - Worsening of symptoms
2. Adverse effects that cause difficulty in continuing the medication

#### Discontinuation of the treatment

The investigator will discontinue the treatment or the study and record the reasons for such when the following conditions occur:

1. Patient withdraws from the study
2. Patient died
3. Patient cannot continue the treatment because of hospital transfer
4. The patient was found to be ineligible for the study
5. Any other cases where the investigator determines that the treatment can be discontinued

#### Endpoints of the study

The primary endpoint of the study is a PSA response rate (i.e., the ratio of patients whose PSA decreased by ≥50% from baseline) at 3 months. Meanwhile, the secondary endpoints in the OCUU-CRPC study are as follows:

1. PSA progression rate at 3 months
2. PSA response rate at 6 months: If initial enzalutamide therapy is switched to other treatments due to disease progression before 6 months, such cases are regarded as “non-responders” regardless of the efficacy of the subsequent treatment. In addition, the PSA response rate in patients in whom flutamide is switched to enzalutamide will be calculated to determine the efficacy of enzalutamide in the flutamide to enzalutamide cohort.
3. PSA progression rate at 6 months
4. Change in quality of life (QOL) as assessed using the Functional Assessment of Cancer Therapy-Prostate (FACT-P) scale in Japanese
5. PSA progression-free survival that is calculated for the initial drug in each arm
6. Adverse events (AEs)

#### Items to record and schedule

1. Patient information during initial diagnosis of prostate cancer including the following:date of birth, diagnosis date, age, PSA level, Gleason score, clinical stage, metastatic sites, and rate of bone metastases
2. Patient information during registration (Table. 1):
  - Patient background data: institution ID, registration date, date of birth, age, height, body weight, PS, concomitant disease, blood sampling (PSA, serum testosterone, white blood cell (WBC), red blood cell (RBC), alkaline phosphatase (ALP), and lactate dehydrogenase (LDH), among others)
  - Imaging: computed tomography (CT) and bone scintigraphy shall be performed on registration to verify metastatic sites
  - QOL (FACT-P)
3. Follow-up after registration (Table. 1);
  - Blood sampling (PSA, WBC, RBC, ALP, and LDH, among others): every month
  - AEs as classified according to the CTCAE ver. 4.0: every month
  - Imaging test: CT and bone scintigraphy when progressive disease is suspected
  - QOL (FACT-P): every 3 months

**Table 1:** Follow-up schedule of examination

	Baseline	1 month	2 months	3 months	Every 1 month	Every 3 months	Discontinuation of therapy
Informed consent	○						
Medical history	○						
Confirmation of eligibility criteria	○						
Randomization	○						
Serum testosterone	○						
FACT-P	○	–	–	○	–	○	○
PSA^a^	○	○	○	○	○	○	○
Biochemical examination^a^	○	○	○	○	○	○	○
CT, bone scintigraphy	○	When progressive disease is suspected^b^					○
Safety	○	○	○	○	○	○	○
Further therapy	–	–	–	Best standard care			

^a^After the discontinuation of enzalutamide or flutamide, PSA and biochemical examination will be continued until September 2018

^b^When progressive disease is suspected, CT and bone scintigraphy will be performed to confirm radiographic disease progression

#### Study period

Registration period: 3 years and 3 months (January 2015–March 2018).

Follow-up period: 6 months after the last patient registration.

#### Ethical consideration and study registration

The study will be performed in accordance with the Declaration of Helsinki and will comply to the International Conference on Harmonization and Good Clinical Practice. All possible treatments and examinations for CRPC are undertaken after obtaining written informed consent from the patients before registration. The OCUU-CRPC study received approval from the institutional ethics committees of the participating institutions. The study has been registered at clinicaltrials.gov (NCT02346578) and the University Hospital Medical Information Network (UMIN000016301).

### Calculation of the target sample size

We hypothesize that the PSA response rate (PSA reduction by ≥50%) of enzalutamide at 3 months is 75%. According to the PREVAIL study [16], the best PSA response rate was 78%. Moreover, most patients in that study achieved a ≥ 50% PSA response at 3 months. Therefore, we can assume 75% as the PSA response rate at 3 months in the enzalutamide arm. On the other hand, the PSA response rate of flutamide is 35% [9]. The primary efficacy endpoint is PSA response rate at 3 months after the initial treatment. A total of 41 patients per group will provide an 90% power to detect an absolute difference in response rate of 50% (75% vs. 25%) at 2-sided α of 0.05 for superiority test with 5% margin. A dropout rate of 10% is expected; thus, *N* = 50 per group is considered to be adequate.

## Discussion

In the PREVAIL study, enzalutamide significantly reduced the risk of radiographic progression and death by 81% (HR: 0.19; *P* < 0.0001) and 29% (HR 0.71, *P* < 0.0001), respectively, compared with a placebo in chemo-naive men with metastatic CRPC [16]. In this study, 61 Japanese were enrolled (enzalutamide, *N* = 28; placebo, *N* = 33), and most them received more than two types of anti-androgens prior to enrollment, including bicalutamide and flutamide. Even after bicalutamide and flutamide, 17 patients (60.7%) had confirmed PSA responses (≥50% reduction from baseline), and enzalutamide reduced the risk of death by 41% (HR: 0.59; 95% CI: 0.20–1.78) [18]. Japanese patients reported less baseline pain, had less soft-tissue disease, and had lower median PSA at baseline. ADT is more widely used as an initial treatment for early stages of prostate cancer in Japan than in other countries [19]. Before the ART era, the treatment options for CRPC were limited, and AAT with flutamide has been widely used in Japan. Even after enzalutamide and abiraterone were introduced, Bic-CAB as primary therapy and AAT with flutamide as the subsequent therapy for advanced prostate cancer were commonly used in Japan.

In the OCUU-CRPC study, we compare the efficacy and safety of enzalutamide and AAT with flutamide in patients with CRPC who were previously treated with CAB therapy with bicalutamide. Because enzalutamide was superior to bicalutamide in terms of PSA response in patients with CRPC who did not receive any prior bicalutamide or chemotherapy in the TERRAIN and STRIVE studies [20, 21], the expected result of the primary endpoint of OCUU-CRPC study will be that enzalutamide is superior to flutamide in terms of PSA response. A longer time to disease progression with enzalutamide than bicalutamide or flutamide may translate to better overall survival, and enzalutamide may be more effective for men with low-volume disease according to post hoc analysis of the PREVAIL trial [22]. However, AAT with flutamide was particularly effective for patients with CRPC who had long response duration to Bic-CAB [8], and the effect of enzalutamide after AAT with flutamide was preserved [18]. Because enzalutamide and flutamide costs $2478 and $208 ($118 if generic drug) per month in Japan, respectively, AAT with flutamide may be more beneficial and accessible for limited patients owing to its lower cost.

The OCUU-CRPC study has some limitations. The PSA response rate at 3 months without radiographic examinations as the primary endpoint may not be associated directly with overall survival. Moreover, this is not a crossover trial and the treatment after enzalutamide and flutamide is not defined by the protocol. Additionally, because the patients included in this study are Japanese and because flutamide is not commonly used for CRPC in other countries, the findings of the OCUU-CRPC study cannot be generalized and applied to patient populations in other locations or regions.

Currently, a similar clinical trial with enzalutamide and flutamide for CRPC (NCT02918968) is undergoing in Japan, in which the primary endpoint is time to PSA progression with first-line therapy. The result of the NCT02918968 will be published a few years after our result.

## Abbreviations

AAT: Alternative antiandrogen therapy
ADT: Androgen-deprivation therapy
AEs: Adverse events
ALP: Alkaline phosphatase
ART: Androgen receptor targeted therapy
AWS: Antiandrogen withdrawal syndrome
CAB Bic: Combined androgen blockade therapy with bicalutamide
CAB: Combined androgen blockade
CRPC: Castration-resistant prostate cancer
CT: Computed tomography
CTCAE: Common Terminology Criteria for Adverse Events
ECOG-PS: Eastern Cooperative Oncology Group performance status
FACT-P: The Functional Assessment of Cancer Therapy-Prostate
LDH: Lactate dehydrogenase
LHRH: Luteinizing hormone-releasing hormone
PSA: Prostate specific antigen
QOL: Quality of life
RBC: Red blood cell
WBC: White blood cell
